# Supplementary figures and images for: Single-Atom Ce-N-C Nanozyme Ameliorates Type 2 Diabetes Mellitus by Improving Glucose Metabolism Disorders and Reducing Oxidative Stress
Source: Biomolecules. 2024 Sep 22;14(9):1193. doi: 10.3390/biom14091193 (PMC11430424; doi:10.3390/biom14091193)

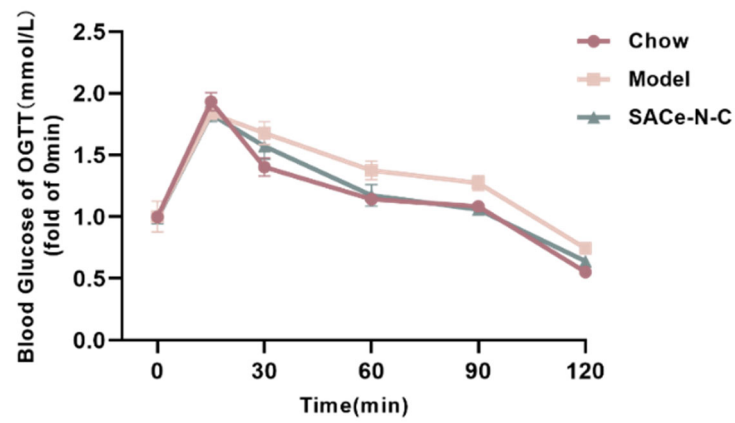

Figure S1. OGTT normalized with zero time point.

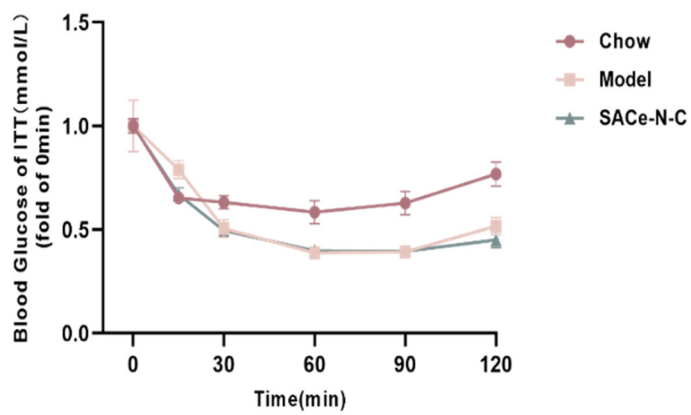

Figure S2. ITT normalized with zero time point.

Supplement: Supplementary file 1 [file biomolecules-14-01193-s001.zip › biomolecules-3138933-supplementary.pdf]
